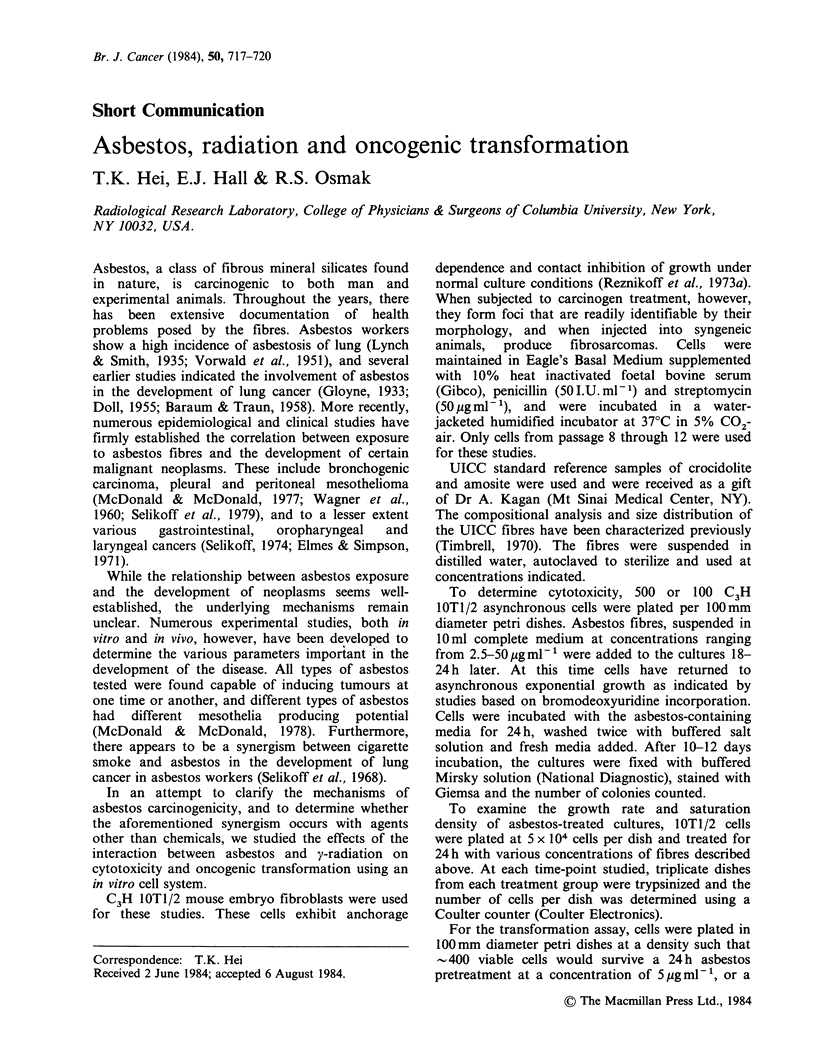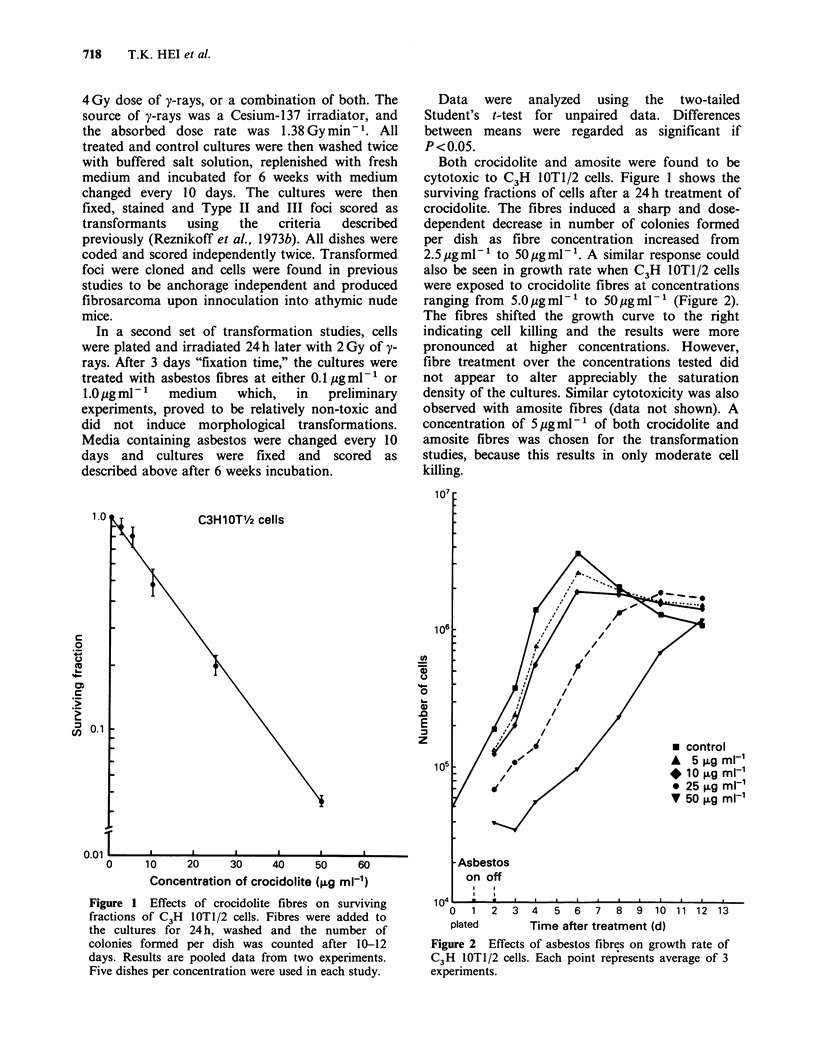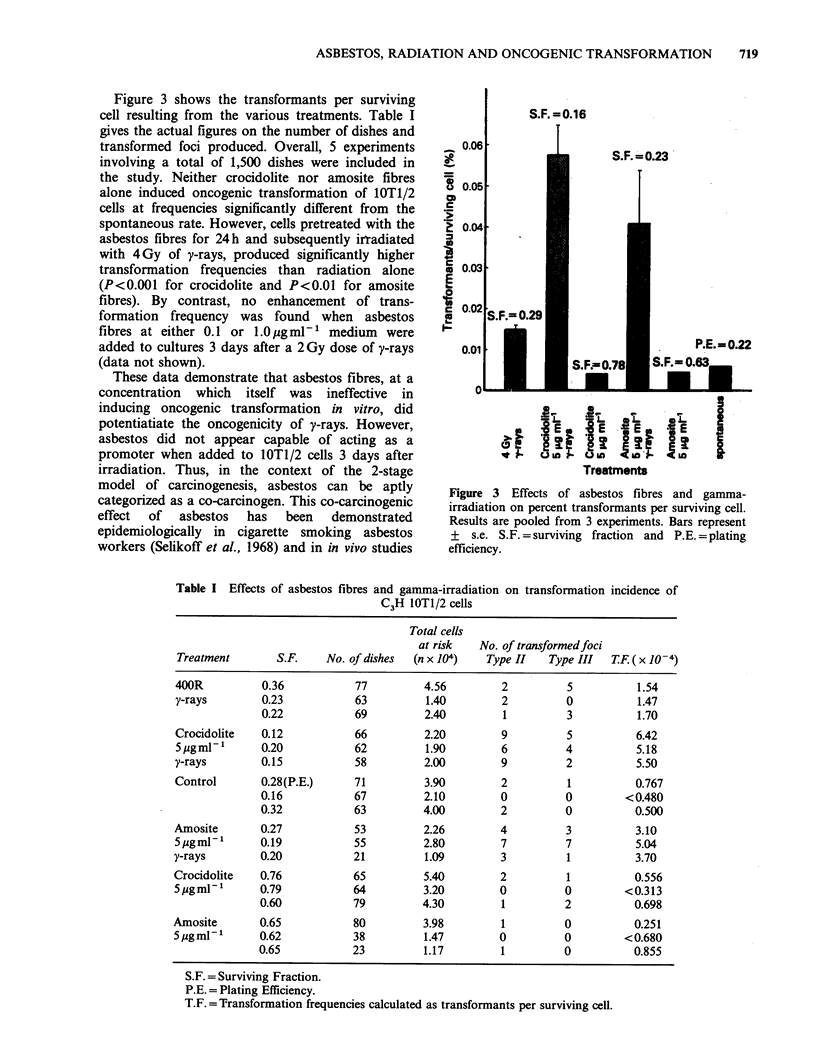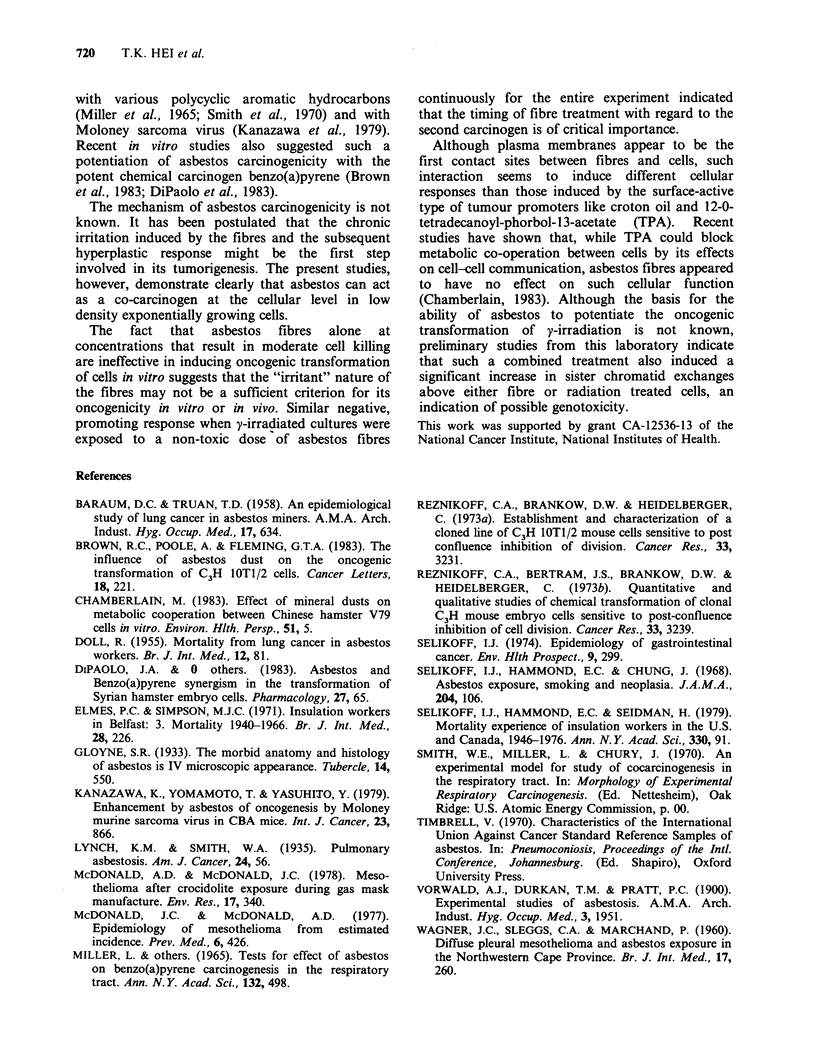# Asbestos, radiation and oncogenic transformation.

**DOI:** 10.1038/bjc.1984.241

**Published:** 1984-11

**Authors:** T. K. Hei, E. J. Hall, R. S. Osmak


Br. J. Cancer (1984), 50, 717–720

### Short Communication

### Asbestos, radiation and oncogenic transformation

T.K. Hei, E.J. Hall & R.S. Osmak

*Radiological Research Laboratory, College of Physicians & Surgeons of Columbia University, New York, NY 10032, USA.*

Asbestos, a class of fibrous mineral silicates found in nature, is carcinogenic to both man and experimental animals. Throughout the years, there has been extensive documentation of health problems posed by the fibres. Asbestos workers show a high incidence of asbestosis of lung (Lynch & Smith, 1935; Vorwald *et al.*, 1951), and several earlier studies indicated the involvement of asbestos in the development of lung cancer (Gloyne, 1933; Doll, 1955; Baraum & Traun, 1958). More recently, numerous epidemiological and clinical studies have firmly established the correlation between exposure to asbestos fibres and the development of certain malignant neoplasms. These include bronchogenic carcinoma, pleural and peritoneal mesothelioma (McDonald & McDonald, 1977; Wagner *et al.*, 1960; Selikoff *et al.*, 1979), and to a lesser extent various gastrointestinal, oropharyngeal and laryngeal cancers (Selikoff, 1974; Elmes & Simpson, 1971).

While the relationship between asbestos exposure and the development of neoplasms seems well-established, the underlying mechanisms remain unclear. Numerous experimental studies, both *in vitro* and *in vivo*, however, have been developed to determine the various parameters important in the development of the disease. All types of asbestos tested were found capable of inducing tumours at one time or another, and different types of asbestos had different mesothelia producing potential (McDonald & McDonald, 1978). Furthermore, there appears to be a synergism between cigarette smoke and asbestos in the development of lung cancer in asbestos workers (Selikoff *et al.*, 1968).

In an attempt to clarify the mechanisms of asbestos carcinogenicity, and to determine whether the aforementioned synergism occurs with agents other than chemicals, we studied the effects of the interaction between asbestos and $\gamma$-radiation on cytotoxicity and oncogenic transformation using an *in vitro* cell system.

$C_3H$ 10T1/2 mouse embryo fibroblasts were used for these studies. These cells exhibit anchorage dependence and contact inhibition of growth under normal culture conditions (Reznikoff *et al.*, 1973*a*). When subjected to carcinogen treatment, however, they form foci that are readily identifiable by their morphology, and when injected into syngeneic animals, produce fibrosarcomas. Cells were maintained in Eagle's Basal Medium supplemented with 10% heat inactivated foetal bovine serum (Gibco), penicillin (50 I.U. $ml^{-1}$) and streptomycin (50 $\mu g\,ml^{-1}$), and were incubated in a water-jacketed humidified incubator at 37°C in 5% $CO_2$-air. Only cells from passage 8 through 12 were used for these studies.

UICC standard reference samples of crocidolite and amosite were used and were received as a gift of Dr A. Kagan (Mt Sinai Medical Center, NY). The compositional analysis and size distribution of the UICC fibres have been characterized previously (Timbrell, 1970). The fibres were suspended in distilled water, autoclaved to sterilize and used at concentrations indicated.

To determine cytotoxicity, 500 or 100 $C_3H$ 10T1/2 asynchronous cells were plated per 100 mm diameter petri dishes. Asbestos fibres, suspended in 10 ml complete medium at concentrations ranging from 2.5–50 $\mu g\,ml^{-1}$ were added to the cultures 18–24 h later. At this time cells have returned to asynchronous exponential growth as indicated by studies based on bromodeoxyuridine incorporation. Cells were incubated with the asbestos-containing media for 24 h, washed twice with buffered salt solution and fresh media added. After 10–12 days incubation, the cultures were fixed with buffered Mirsky solution (National Diagnostic), stained with Giemsa and the number of colonies counted.

To examine the growth rate and saturation density of asbestos-treated cultures, 10T1/2 cells were plated at $5\times10^4$ cells per dish and treated for 24 h with various concentrations of fibres described above. At each time-point studied, triplicate dishes from each treatment group were trypsinized and the number of cells per dish was determined using a Coulter counter (Coulter Electronics).

For the transformation assay, cells were plated in 100 mm diameter petri dishes at a density such that $\sim$400 viable cells would survive a 24 h asbestos pretreatment at a concentration of 5 $\mu g\,ml^{-1}$, or a

Correspondence: T.K. Hei
Received 2 June 1984; accepted 6 August 1984.

© The Macmillan Press Ltd., 1984

4 Gy dose of γ-rays, or a combination of both. The source of γ-rays was a Cesium-137 irradiator, and the absorbed dose rate was 1.38 Gy $min^{-1}$. All treated and control cultures were then washed twice with buffered salt solution, replenished with fresh medium and incubated for 6 weeks with medium changed every 10 days. The cultures were then fixed, stained and Type II and III foci scored as transformants using the criteria described previously (Reznikoff *et al.*, 1973*b*). All dishes were coded and scored independently twice. Transformed foci were cloned and cells were found in previous studies to be anchorage independent and produced fibrosarcoma upon innoculation into athymic nude mice.

In a second set of transformation studies, cells were plated and irradiated 24 h later with 2 Gy of γ-rays. After 3 days "fixation time," the cultures were treated with asbestos fibres at either 0.1 μg $ml^{-1}$ or 1.0 μg $ml^{-1}$ medium which, in preliminary experiments, proved to be relatively non-toxic and did not induce morphological transformations. Media containing asbestos were changed every 10 days and cultures were fixed and scored as described above after 6 weeks incubation.

Data were analyzed using the two-tailed Student's *t*-test for unpaired data. Differences between means were regarded as significant if $P < 0.05$.

Both crocidolite and amosite were found to be cytotoxic to $C_3H$ 10T1/2 cells. Figure 1 shows the surviving fractions of cells after a 24 h treatment of crocidolite. The fibres induced a sharp and dose-dependent decrease in number of colonies formed per dish as fibre concentration increased from 2.5 μg $ml^{-1}$ to 50 μg $ml^{-1}$. A similar response could also be seen in growth rate when $C_3H$ 10T1/2 cells were exposed to crocidolite fibres at concentrations ranging from 5.0 μg $ml^{-1}$ to 50 μg $ml^{-1}$ (Figure 2). The fibres shifted the growth curve to the right indicating cell killing and the results were more pronounced at higher concentrations. However, fibre treatment over the concentrations tested did not appear to alter appreciably the saturation density of the cultures. Similar cytotoxicity was also observed with amosite fibres (data not shown). A concentration of 5 μg $ml^{-1}$ of both crocidolite and amosite fibres was chosen for the transformation studies, because this results in only moderate cell killing.

**Figure 1** Effects of crocidolite fibres on surviving fractions of $C_3H$ 10T1/2 cells. Fibres were added to the cultures for 24 h, washed and the number of colonies formed per dish was counted after 10–12 days. Results are pooled data from two experiments. Five dishes per concentration were used in each study.

**Figure 2** Effects of asbestos fibres on growth rate of $C_3H$ 10T1/2 cells. Each point represents average of 3 experiments.

Figure 3 shows the transformants per surviving cell resulting from the various treatments. Table I gives the actual figures on the number of dishes and transformed foci produced. Overall, 5 experiments involving a total of 1,500 dishes were included in the study. Neither crocidolite nor amosite fibres alone induced oncogenic transformation of 10T1/2 cells at frequencies significantly different from the spontaneous rate. However, cells pretreated with the asbestos fibres for 24 h and subsequently irradiated with 4 Gy of γ-rays, produced significantly higher transformation frequencies than radiation alone ($P<0.001$ for crocidolite and $P<0.01$ for amosite fibres). By contrast, no enhancement of transformation frequency was found when asbestos fibres at either 0.1 or 1.0 $\mu g\,ml^{-1}$ medium were added to cultures 3 days after a 2 Gy dose of γ-rays (data not shown).

These data demonstrate that asbestos fibres, at a concentration which itself was ineffective in inducing oncogenic transformation *in vitro*, did potentiatiate the oncogenicity of γ-rays. However, asbestos did not appear capable of acting as a promoter when added to 10T1/2 cells 3 days after irradiation. Thus, in the context of the 2-stage model of carcinogenesis, asbestos can be aptly categorized as a co-carcinogen. This co-carcinogenic effect of asbestos has been demonstrated epidemiologically in cigarette smoking asbestos workers (Selikoff *et al.*, 1968) and in *in vivo* studies

**Figure 3** Effects of asbestos fibres and gamma-irradiation on percent transformants per surviving cell. Results are pooled from 3 experiments. Bars represent ± s.e. S.F. = surviving fraction and P.E. = plating efficiency.

**Table I** Effects of asbestos fibres and gamma-irradiation on transformation incidence of $C_3H$ 10T1/2 cells

| *Treatment* | *S.F.* | *No. of dishes* | *Total cells at risk* $(n \times 10^4)$ | *No. of transformed foci Type II* | *Type III* | *T.F.* $(\times 10^{-4})$ |
|---|---|---|---|---|---|---|
| 400R | 0.36 | 77 | 4.56 | 2 | 5 | 1.54 |
| γ-rays | 0.23 | 63 | 1.40 | 2 | 0 | 1.47 |
| | 0.22 | 69 | 2.40 | 1 | 3 | 1.70 |
| Crocidolite | 0.12 | 66 | 2.20 | 9 | 5 | 6.42 |
| 5 $\mu g\,ml^{-1}$ | 0.20 | 62 | 1.90 | 6 | 4 | 5.18 |
| γ-rays | 0.15 | 58 | 2.00 | 9 | 2 | 5.50 |
| Control | 0.28(P.E.) | 71 | 3.90 | 2 | 1 | 0.767 |
| | 0.16 | 67 | 2.10 | 0 | 0 | <0.480 |
| | 0.32 | 63 | 4.00 | 2 | 0 | 0.500 |
| Amosite | 0.27 | 53 | 2.26 | 4 | 3 | 3.10 |
| 5 $\mu g\,ml^{-1}$ | 0.19 | 55 | 2.80 | 7 | 7 | 5.04 |
| γ-rays | 0.20 | 21 | 1.09 | 3 | 1 | 3.70 |
| Crocidolite | 0.76 | 65 | 5.40 | 2 | 1 | 0.556 |
| 5 $\mu g\,ml^{-1}$ | 0.79 | 64 | 3.20 | 0 | 0 | <0.313 |
| | 0.60 | 79 | 4.30 | 1 | 2 | 0.698 |
| Amosite | 0.65 | 80 | 3.98 | 1 | 0 | 0.251 |
| 5 $\mu g\,ml^{-1}$ | 0.62 | 38 | 1.47 | 0 | 0 | <0.680 |
| | 0.65 | 23 | 1.17 | 1 | 0 | 0.855 |

S.F. = Surviving Fraction.
P.E. = Plating Efficiency.
T.F. = Transformation frequencies calculated as transformants per surviving cell.

with various polycyclic aromatic hydrocarbons (Miller *et al.*, 1965; Smith *et al.*, 1970) and with Moloney sarcoma virus (Kanazawa *et al.*, 1979). Recent *in vitro* studies also suggested such a potentiation of asbestos carcinogenicity with the potent chemical carcinogen benzo(a)pyrene (Brown *et al.*, 1983; DiPaolo *et al.*, 1983).

The mechanism of asbestos carcinogenicity is not known. It has been postulated that the chronic irritation induced by the fibres and the subsequent hyperplastic response might be the first step involved in its tumorigenesis. The present studies, however, demonstrate clearly that asbestos can act as a co-carcinogen at the cellular level in low density exponentially growing cells.

The fact that asbestos fibres alone at concentrations that result in moderate cell killing are ineffective in inducing oncogenic transformation of cells *in vitro* suggests that the "irritant" nature of the fibres may not be a sufficient criterion for its oncogenicity *in vitro* or *in vivo*. Similar negative, promoting response when γ-irradiated cultures were exposed to a non-toxic dose of asbestos fibres continuously for the entire experiment indicated that the timing of fibre treatment with regard to the second carcinogen is of critical importance.

Although plasma membranes appear to be the first contact sites between fibres and cells, such interaction seems to induce different cellular responses than those induced by the surface-active type of tumour promoters like croton oil and 12-0-tetradecanoyl-phorbol-13-acetate (TPA). Recent studies have shown that, while TPA could block metabolic co-operation between cells by its effects on cell–cell communication, asbestos fibres appeared to have no effect on such cellular function (Chamberlain, 1983). Although the basis for the ability of asbestos to potentiate the oncogenic transformation of γ-irradiation is not known, preliminary studies from this laboratory indicate that such a combined treatment also induced a significant increase in sister chromatid exchanges above either fibre or radiation treated cells, an indication of possible genotoxicity.

This work was supported by grant CA-12536-13 of the National Cancer Institute, National Institutes of Health.

**References**

BARAUM, D.C. & TRUAN, T.D. (1958). An epidemiological study of lung cancer in asbestos miners. A.M.A. Arch. Indust. *Hyg. Occup. Med.*, **17**, 634.

BROWN, R.C., POOLE, A. & FLEMING, G.T.A. (1983). The influence of asbestos dust on the oncogenic transformation of $C_3H$ 10T1/2 cells. *Cancer Letters*, **18**, 221.

CHAMBERLAIN, M. (1983). Effect of mineral dusts on metabolic cooperation between Chinese hamster V79 cells *in vitro*. *Environ. Hlth. Persp.*, **51**, 5.

DOLL, R. (1955). Mortality from lung cancer in asbestos workers. *Br. J. Int. Med.*, **12**, 81.

DIPAOLO, J.A. & 0 others. (1983). Asbestos and Benzo(a)pyrene synergism in the transformation of Syrian hamster embryo cells. *Pharmacology*, **27**, 65.

ELMES, P.C. & SIMPSON, M.J.C. (1971). Insulation workers in Belfast: 3. Mortality 1940–1966. *Br. J. Int. Med.*, **28**, 226.

GLOYNE, S.R. (1933). The morbid anatomy and histology of asbestos is IV microscopic appearance. *Tubercle*, **14**, 550.

KANAZAWA, K., YOMAMOTO, T. & YASUHITO, Y. (1979). Enhancement by asbestos of oncogenesis by Moloney murine sarcoma virus in CBA mice. *Int. J. Cancer*, **23**, 866.

LYNCH, K.M. & SMITH, W.A. (1935). Pulmonary asbestosis. *Am. J. Cancer*, **24**, 56.

MCDONALD, A.D. & MCDONALD, J.C. (1978). Mesothelioma after crocidolite exposure during gas mask manufacture. *Env. Res.*, **17**, 340.

MCDONALD, J.C. & MCDONALD, A.D. (1977). Epidemiology of mesothelioma from estimated incidence. *Prev. Med.*, **6**, 426.

MILLER, L. & others. (1965). Tests for effect of asbestos on benzo(a)pyrene carcinogenesis in the respiratory tract. *Ann. N.Y. Acad. Sci.*, **132**, 498.

REZNIKOFF, C.A., BRANKOW, D.W. & HEIDELBERGER, C. (1973*a*). Establishment and characterization of a cloned line of $C_3H$ 10T1/2 mouse cells sensitive to post confluence inhibition of division. *Cancer Res.*, **33**, 3231.

REZNIKOFF, C.A., BERTRAM, J.S., BRANKOW, D.W. & HEIDELBERGER, C. (1973*b*). Quantitative and qualitative studies of chemical transformation of clonal $C_3H$ mouse embryo cells sensitive to post-confluence inhibition of cell division. *Cancer Res.*, **33**, 3239.

SELIKOFF, I.J. (1974). Epidemiology of gastrointestinal cancer. *Env. Hlth Prospect.*, **9**, 299.

SELIKOFF, I.J., HAMMOND, E.C. & CHUNG, J. (1968). Asbestos exposure, smoking and neoplasia. *J.A.M.A.*, **204**, 106.

SELIKOFF, I.J., HAMMOND, E.C. & SEIDMAN, H. (1979). Mortality experience of insulation workers in the U.S. and Canada, 1946–1976. *Ann. N.Y. Acad. Sci.*, **330**, 91.

SMITH, W.E., MILLER, L. & CHURY, J. (1970). An experimental model for study of cocarcinogenesis in the respiratory tract. In: *Morphology of Experimental Respiratory Carcinogenesis*. (Ed. Nettesheim), Oak Ridge: U.S. Atomic Energy Commission, p. 00.

TIMBRELL, V. (1970). Characteristics of the International Union Against Cancer Standard Reference Samples of asbestos. In: *Pneumoconiosis, Proceedings of the Intl. Conference, Johannesburg*. (Ed. Shapiro), Oxford University Press.

VORWALD, A.J., DURKAN, T.M. & PRATT, P.C. (1900). Experimental studies of asbestosis. A.M.A. Arch. Indust. *Hyg. Occup. Med.*, **3**, 1951.

WAGNER, J.C., SLEGGS, C.A. & MARCHAND, P. (1960). Diffuse pleural mesothelioma and asbestos exposure in the Northwestern Cape Province. *Br. J. Int. Med.*, **17**, 260.